# Machine learning performance in a microbial molecular autopsy context: A cross-sectional postmortem human population study

**DOI:** 10.1371/journal.pone.0213829

**Published:** 2019-04-15

**Authors:** Yu Zhang, Jennifer L. Pechal, Carl J. Schmidt, Heather R. Jordan, Wesley W. Wang, M. Eric Benbow, Sing-Hoi Sze, Aaron M. Tarone

**Affiliations:** 1 Texas A&M University, Department of Computer Science and Engineering, College Station, TX, United States of America; 2 Michigan State University, Department of Entomology, East Lansing, MI, United States of America; 3 Wayne County Medical Examiner’s Office, Detroit, MI, United States of America; 4 University of Michigan, Department of Pathology, Ann Arbor, MI, United States of America; 5 Mississippi State University, Department of Biological Sciences, Mississippi State, MS, United States of America; 6 Texas A&M University, Department of Chemistry, College Station, TX, United States of America; 7 Michigan State University, Department of Osteopathic Medical Specialties, East Lansing, MI, United States of America; 8 Michigan State University, Ecology, Evolutionary Biology, and Behavior Program, East Lansing, MI, United States of America; 9 Texas A&M University, Department of Biochemistry and Biophysics, College Station, TX, United States of America; 10 Texas A&M University, Department of Entomology, College Station, TX, United States of America; Zhejiang University, CHINA

## Abstract

**Background:**

The postmortem microbiome can provide valuable information to a death investigation and to the human health of the once living. Microbiome sequencing produces, in general, large multi-dimensional datasets that can be difficult to analyze and interpret. Machine learning methods can be useful in overcoming this analytical challenge. However, different methods employ distinct strategies to handle complex datasets. It is unclear whether one method is more appropriate than others for modeling postmortem microbiomes and their ability to predict attributes of interest in death investigations, which require understanding of how the microbial communities change after death and may represent those of the once living host.

**Methods and findings:**

Postmortem microbiomes were collected by swabbing five anatomical areas during routine death investigation, sequenced and analyzed from 188 death cases. Three machine learning methods (boosted algorithms, random forests, and neural networks) were compared with respect to their abilities to predict case attributes: postmortem interval (PMI), location of death, and manner of death. Accuracy depended on the method used, the numbers of anatomical areas analyzed, and the predicted attribute of death.

**Conclusions:**

All algorithms performed well but with distinct features to their performance. Xgboost often produced the most accurate predictions but may also be more prone to overfitting. Random forest was the most stable across predictions that included more anatomic areas. Analysis of postmortem microbiota from more than three anatomic areas appears to yield limited returns on accuracy, with the eyes and rectum providing the most useful information correlating with circumstances of death in most cases for this dataset.

## Introduction

The microbiome is comprised of all the microbes within a host, space, or community that represent complex consortia of many species [1]. The composition and role of microbial consortia that endogenously and exogenously comprise the human microbiome has been extensively studied for human health [2,3]. These communities are known to be variable within an individual, as distinct consortia reside on or in different parts of the body (e.g., the gut, skin, eyes, mouth) in a way that represents a dynamic ecosystem [4]. Additionally, the clinical importance of the human microbiome is influenced by the host environment, presence or absence of disease, development, and lifestyle markers, such as prescribed or illicit chemical substances and nutrition [2,3]. Little is known, however, about microbial biodiversity dynamics in human populations after death [5] until our recent discovery that the postmortem microbiota (i.e., bacteria and archeae) was consistent until 48 h after death, when the communities were found to significantly change in composition [6]. This study showed data with potential application in forensic science, but also demonstrated that there were microbial signatures associated with health status of the once living hosts, but only within a two-day postmortem interval. These data may be valuable resources for understanding the antemortem health from individuals where antemortem samples are difficult to obtain. Additionally, these samples may have predictive value for health states within populations. Despite this potential to use the postmortem microbiota, refinement of computation analyses and predictive data modeling will be necessary to understand the full value of this information.

Eventually, all hosts cease living and the natural processes of decomposition will take over. The body provides some of the most critical information for estimating a time since death, manner of death or event location of death based on the biological and chemical changes that occur during decomposition [7]. There are many biological and chemical changes after death, such as rigor mortis and algor mortis, although there are compartments, such as the vitreous humor, that may take longer to be affected by the changes due to decomposition. As with most biological markers there are potential confounding factors, such as clothing, body mass and environment, which affect the ability to make postmortem diagnostic and forensic assessments, e.g., diagnosis of disease or how much time has elapsed since death. In particular, a paucity of data exists about the succession of microflora residing within the human ecosystem, their associations with decomposition, and the potential widespread use in clinical and forensic applications as a minimally invasive autopsy technique. Part of understanding the postmortem microbiome for potential use in forensic investigation is determining how these microbial communities vary among anatomical areas and how they change after death. Given the well-documented importance of the microbiome to the living host, the use of the postmortem microbiome, if it resembles the live host microbiome, also holds promise for a robust and widespread method of public health surveillance, which we have recently shown [6].

While studies of the microbiota for use during death investigation have undergone a rapid increase in the past five years as reviewed in Pechal et al [6]; these studies have often resulted from small-scale data (n < 55 cases) collected at anthropological research facilities, which occur in distinct, well documented conditions not encumbered by the variability and challenging circumstances involved with routine case work. For a more informed understanding of how postmortem microbiomes can be used in death investigations it will be important to evaluate these methods in the context of actual cases. To the best of our knowledge, our collaborative study was the only available that employed a large-scale cross-sectional survey of postmortem microbiomes (n = 188 cases) and showed significant differences in postmortem microbiota among anatomic locations and changes over time since death [6]. Thus, there is potential for the use of native postmortem microbial communities, easily collected with minimal training, to serve as a valuable adjunct to the autopsy process or to collect information when it is not practical to perform an autopsy.

Molecular autopsies have primarily been used as a diagnostic tool in cases with unremarkable pathological findings to identify gene mutations that may have resulted in a sudden or unexplained death. Advances in technologies have demonstrated that high-throughput genomic technologies could also be used to characterize and analyze microbiota associated with the once living and, afterward, the cause or manner of death [5,6,8,9]. Resulting datasets from high-throughput sequencing of microbial communities often represent complex datasets that are not amenable to analysis with non-machine learning methods. By and large, the analysis of postmortem microbial data has been limited to two major types of machine learning methods–random forests and gradient boosting [6,10–12], though it is not clear if these are the best algorithms for analyzing the data or if there is a best machine learning approach for making predictions from postmortem microbiomes.

This computational challenge is not restricted to forensic microbiology. In an evaluation of hyperspectral imaging for remote sensing in ecology research, random forests and boosted methods performed similarly well (~70% accurate) and outperformed neural network (~64% accurate) [13]. A similar pattern was observed with “electronic tongue” analyses, which was attributed to the ability of random forests to better handle unbalanced data and small sample sizes compared to neural network [14]. However, boosted methods can sometimes outperform random forests [15]. This difference in performance comes with the tradeoff of being less generalizable as the methods can be prone to overfitting due to the weighting strategy used in their development [16]. In an investigative context this feature could detract from its usefulness in application and may also guide decisions regarding the use of smaller local databases or larger regional / national databases. It is also unclear whether certain types of samples (e.g., microbial communities from different parts of the body) are more informative than others for postmortem predictions or provide different value to different methods. Thus, it is an open question if certain types of methods and samples are most effective for postmortem estimations and to evaluate the use of microbial communities in medical diagnoses.

One challenge that is distinct to forensic investigations and for the future of forensic microbiology is the need for an understanding of error in predictions. In the United States of America, the presentation of scientific evidence in the courtroom is generally bound by the Daubert standard [17], which in part requires knowledge of error in any scientific opinion provided to a court. Additionally, in 2009 the National Research Council [18] issued a report highlighting the need to improve basic research in forensic science. With respect to the challenge of making predictions with machine learning methods, these perspectives are particularly important. These methods are essential to assessing large and multi-dimensional datasets. However, the complexity of these algorithms means that they are effectively “black boxes” where information goes into the black box, something happens in the black box, and a prediction comes out of the black box. In such an instance, it is possible for non-intuitive results to occur. The most obvious of these problems is overfitting of a model, where a methods develops an excellent predictor from one dataset, which by all indications should perform well with new data, but that model is too specific to the features of its parent dataset and fails to effectively predict in a generalizable fashion. There is nothing inherently wrong with such an approach, as multi-dimensional datasets generally defy solutions that are intuitive to human understanding. However, from legal and practical perspectives, this feature means that a careful evaluation of the qualities of methods employed is required in order to ensure the quality of postmortem information provided to a death investigation.

Machine learning methods using microbial signatures to predict time, manner, location of death and associations with medical conditions in the living have remained limited. Identification of robust microbial based biomarkers using supervised learning algorithms could yield a fruitful and underutilized molecular autopsy approach during routine death investigation and for future clinical diagnostic uses. However, there are a variety of algorithms that could be implemented with microbial datasets and it remains unclear if there is an advantage to using one over the others in datasets that represent the typical size and structure encountered in postmortem microbiology.

Here, we aimed to evaluate machine learning methods performance of microbial community analyses developed from a large-scale survey of postmortem samples during routine casework. This analytical evaluation is a first step toward developing guidelines for the use of microbial databases in death investigations, and for potential utility in medical diagnostics. We approached the problem from a computer science perspective, using one of the few datasets that can provide sufficient power for evaluating machine learning methods for this purpose. Our goal is to begin the process of determining best practices for conducting machine learning-based predictions with microbial datasets for medical and forensic purposes. We assess the outcomes of three machine learning methods–random forests, neural network, and boosted algorithms [19–21]; to understand their relative performances when making predictions of our target death investigation interests using microbial sequence information derived from one to five swabs taken to represent microbial communities from different anatomical areas. The goal of this endeavor is to develop an understanding for how many samples must be taken to provide useful investigative predictions, which anatomical areas may provide the most information for these purposes, how useful such predictions will be, determine if different algorithms identify similar or different informative variables, and which algorithms are most likely to perform best for these purposes.

## Materials and methods

Two major datasets were used for this research. The first dataset contains all the meta-information of the samples derived from previously published research on the postmortem microbiome, henceforth referred to as “metadata” [6]. The metadata include the sample area (where on the body a microbial sample was collected), sex, race, age, death location (in a hospital, indoors, outdoors), estimated postmortem interval (reported in temporal blocks), manner of death (suicide, homicide, etc.), season of death, body mass index (BMI), and weight status (obese, etc.). Samples were obtained during routine death investigation at the Wayne County Medical Examiner’s Office (Detroit, Michigan), as previously described in [6]. Briefly, this was done by swabbing anatomic regions of interest with DNA-Free sterile cotton-tipped applicators (Puritan). The region of interest was physically rubbed while rotating for 3–5 seconds, then the tip of the applicator was placed into a sterile microcentrifuge tube (VWR) filled with 200 μL of 100% molecular grade ethanol (Fisher Scientific). These were stored at -20°C for further processing. Genomic DNA was isolated with the PureLink Genomic DNA Mini kit.

The other dataset is the microbial taxonomic information obtained through targeted high-throughput 16S rRNA gene amplicon sequencing (Illumina MiSeq), which is described in detail in Pechal et al. [6] and can be found archived through the European Bioinformatics Institute European Nucleotide Archive (www.ebi.ac.uk/ena) under accession number: PRJEB22642. We cleaned the two datasets separately and then merged them into one dataset (*n* = 188 cases, S1 Data, S2 Data), such that each row contains both metadata and microbial taxon data (phylum, class, order, family, genus, species, operational taxonomic unit) for each sample. We built models using three machine learning methods (xgboost, random forest, and neural network) [19–21], with each method applied to predict attributes of the case: the postmortem interval, manner of death, and location of death.

Among the three methods, both “xgboost” and “random forest” are tree-based algorithms. Tree-based methods use splitting rules for classification or regression. Usually a single tree does not provide competitive accuracy, but combining multiple trees into one consensus prediction can help improve performance, and reduce variance [22]. Random forest methods resample a random portion of all predictors and get one tree for each iteration and combine them together. “xgboost”, short for “Extreme Gradient Boosting”, uses a boosted trees algorithm. It grows sequentially to a single tree by fitting on residuals. Instead of sampling original data, it fits modified data (residuals) after each split in the tree, effectively weighing subsequent splits in the tree by their ability to provide information independent of information provided by previous splits in the tree [23]. Both of these methods are frequently used in machine learning applications. Xgboost is gaining more attention since it has shown good performance in many data science competitions. In the biological disciplines, random forest is more frequently used, but without a clear reason. Another common type of machine learning algorithm employed to solve problems with multi-dimensional datasets is “neural network”. A neural network is a multi-stage regression or classification process. Each layer extracts linear combinations of the previous layer’s inputs as derived features. This makes it a very powerful nonlinear model [24]. However, the potential number of parameters that must be learned by the algorithms can make them unsuitable for small datasets. It has very good performance in fields like graphics and big data analysis.

### Details of implementation

Among each prediction (the postmortem interval, manner of death, and location of death), we first tried to optimize the parameters for each machine learning method. We implemented the parameter optimization in the way of grid search and measured the performance through 5-fold cross validation. After some research, we manually determined a subset of parameter space for each machine learning method. Then the total data were randomly and evenly separated into 5 shares. Then we iteratively trained the model on 4 folds while using the remaining fold as the test set. Then the average of 5 test accuracies was calculated and compared to that of other parameter set. The parameter set having the highest average accuracy was chosen as the set of optimal parameter. The tuned parameters for each prediction and each learning method was displayed in S1 Table. This type of cross validation, is considered to eliminate or limit the over fitting issue noted above. The averaged accuracy, confusion matrix, and evaluation statistics obtained based on cross validation method can also be used as a good metrics to compare across different machine learning methods performance as to the current dataset. We further trained the model on the whole dataset with the tuned parameters to get the important features for each prediction and report comparisons based on these tuned models. For the xgboost method, we used the “0.4.3” version of the “xgboost” package in R [21]. The objective function used for a training model is “multi:softprob”, which is better for datasets with multiple classes. Accordingly, the number of classes is set to “4” as we have four classes of predictions for each question of interest (postmortem interval: < 24 h, 25–48 h, 49–72 h, > 73 h; event location: hospital, indoors, outdoors, vehicular; and manner of death: accident, homicide, natural, suicide). We used the evaluation metric of “mlogloss”. The models with all swabs used the optimal parameter set, but for the combinations of different anatomic areas, the training iterations were adjusted according to the number of anatomic areas in each model. We implemented the random forest method with “4.6.12” version of the “randomForest” R package [19]. For the neural network method, we implemented it with “7.3.12” version of the “nnet” R package [25]. The neural network method also requires converting the target predictor variable to be a factor. Data and code for implementation of each algorithm can be found in S1 Data and S2 Data, respectively.

The following performance metrics within a class for each machine learning methods was initially evaluated with models that included all anatomic areas: sensitivity, specificity, positive predictive value, negative predictive value, prevalence, detection rate, detection prevalence, and balanced accuracy. However, one challenge in analyzing casework with bacterial information is understanding which anatomical area(s) will be most informative for death investigation. We addressed this challenge by evaluating the performances of all three machine learning methods when predicting the attributes of the cases using the postmortem microbiota from different number of anatomic areas; models were built using a greedy algorithm that added anatomic areas that adds most to the previous model accuracy, one by one, for each predictor variable (i.e., postmortem interval, event location, and manner of death). Next, to identify important microbial features (e.g., genus or family taxon) or metadata features (e.g., age, race, sex) resulting from each machine learning method, we selected several of the top features from each method (number of features varied based on method) to plot these potential microbial biomarkers for each predictor attribute (postmortem interval: < 24 h, 25–48 h, 49–72 h, > 73 h; event location: hospital, indoors, outdoors, vehicular; and manner of death: accident, homicide, natural, suicide). Finally, due to the zero-heavy nature of high-throughput sequencing, we compared ratios of non-zero microbial taxa to all microbial taxa. Paired Wilcox sum ranked tests (false discovery rate adjusted p-values) were used to statistically evaluate the top features across the predictor attributes with the “0.9-69-3” version of the “RVAideMemoire” R package [26].

### Human subjects/study population

Institutional Review Board (IRB) review is not required for research on deceased individuals. Health Insurance Portability and Accountability Act of 1996 (HIPAA) is not a consideration after death in the medicolegal context and so there are no privacy issues associated with microbial sampling of bodies during investigation and autopsy. Additionally, human tissue was not intentionally sampled or removed. Microbiological sampling is an established procedure for the diagnosis of pathologies.

## Results

Among all the cases, 44.1% were female and 55.9% were male. The age of the cases ranged from 18–88 years, with a mean of 43.9 and a median of 43.0. For the estimated postmortem interval, 45.7% of the cases had a postmortem interval less than 24h, 41.0% of the cases had a postmortem interval of 25-48h. The postmortem interval of 49-72h and greater than 73h occupied 7.4% and 5.9% of the data respectively. For the event of location, 12.8% were in a hospital, 68.6% were indoors, 12.8% were outdoors, and 5.9% were vehicular. As for the manner of death, 37.8% had an accident, 19.7% were a homicide, 30.3% were natural, and 12.2% were suicide.

### Performance comparison among three prediction methods

To compare the performance of the different machine learning methods, we selected three important attributes during death investigation—postmortem interval, manner of death, and death event location–to predict using the postmortem microbiota. The accuracy achieved by each method varied from 70.6–87.6% (Tables 1 and 2); the xgboost method consistently resulted in the highest accuracy (74.5%– 87.6%) across all predictor variables, with neural network (70.7–83.0%) and random forest (73.6–86.3%) performing comparably. All prediction algorithms exhibited statistically significant models for all prediction types (p-values < 2.0e-16). Based on additional evaluation statistics (Table 3), such as sensitivity, specificity, prevalence, we found that xgboost or random forest tend to perform more favorably than neural network. The positive prediction value using xgboost ranged from 0.73–0.86 for the postmortem interval classes (< 24 h, 25–48 h, 49–72 h, > 73 h); 0.73–1.0 for event location classes (hospital, indoors, outdoors, vehicular); and 0.78–0.93 for manner of death classes (accident, homicide, natural, suicide). Random forest ranged from 0.71–0.94, 0.83–1.0, and 0.75–0.96 in the same comparisons, respectively. In most cases, neural networks underperformed the positive predict values of the other two algorithms, but often by only a few percent (e.g. within 3% for two of the three prediction types in Table 2). While many metrics were comparable across algorithms, there were observable differences in sensitivity, specificity, detection rate, detection prevalence, and balanced accuracy depending on the algorithm and prediction class. The overall picture arising from these comparisons in this database would suggest that xgboost is sometimes more effective than random forest, but is often very comparable, and in some instances will underperform (e.g. PMI estimates with 2 or 3 sample areas included). In most cases neural networks underperform, but there are specific instances where this algorithm exhibited the most balanced accuracy (Table 3, manner of death–vehicular deaths). Likewise, random forest was often comparable to xgboost, but could underperform both competing algorithms in specific instances (Table 3, PMI > 48 hours).

**Table 1:** Confusion matrices for prediction of postmortem interval, event location, and manner of death with the microbiota from all anatomic locations (ears, eyes, nose, mouth, and rectum) using three machine learning methods: xgboost, random forest, and neural network. The results for the three methods are put within the same table in the order of xgboost/ random forest/ neural network.

Predictor Variable	prediction/ observation	< 24 h	25–48 h	49–72 h	> 73 h
**Postmortem Interval Estimate**	**< 24 h**	296/ 300/ 285	78/ 63/ 71	19/ 28/ 22	07/ 06/ 05
	**25–48 h**	79/ 76/ 78	271/288/268	11/ 18/ 20	11/ 26/ 15
	**49–72 h**	04/ 03/ 13	02/ 02/ 08	34/ 20/ 20	00/ 01/ 03
	**> 73 h**	01/ 01/ 04	02/ 00/ 06	02/ 00/ 04	30/ 15/ 25
	**prediction/** **observation**	**Hospital**	**Indoors**	**Outdoors**	**Vehicle**
**Event Location**	**Hospital**	75/ 65/ 69	03/ 00/ 19	05/ 00/ 07	03/ 00/ 07
	**Indoors**	31/ 42/ 30	580/ 586/ 544	25/ 39/ 22	14/ 19/ 09
	**Outdoors**	05/ 05/ 05	05/ 03/ 22	75/ 67/ 72	11/ 06/ 06
	**Vehicle**	01/ 00/ 08	01/ 00/ 04	01/ 00/ 05	12/ 13/ 18
	**prediction/** **observation**	**Accident**	**Homicide**	**Natural**	**Suicide**
**Manner of Death**	**Accident**	282/296/266	12/ 11/ 26	29/ 18/ 51	27/ 38/ 27
	**Homicide**	07/ 03/ 17	142/ 141/ 120	04/ 03/ 11	00/ 00/ 12
	**Natural**	42/ 37 /42	03/ 09/ 09	211/ 221/169	14/ 26/ 20
	**Suicide**	06/ 01 / 12	05/ 01/ 07	01/ 03 / 14	62/ 39/ 44

**Table 2:** Accuracy and p-value obtained from 5-fold cross validation for three machine learning methods (xgboost, random forest and neural network) for the prediction of postmortem interval, event location and manner of death using the microbiota from all anatomic locations (ears, eyes, nose, mouth, and rectum).

Predictor Variable	Performance Metric	xgboost	random forest	neural network
**Postmortem Interval**	Accuracy	0.745	0.736	0.706
	p-value	< 2.0e-16	< 2.0e-16	< 2.0e-16
**Event Location**	Accuracy	0.876	0.863	0.830
	p-value	< 2.0e-16	< 2.0e-16	< 2.0e-16
**Manner of Death**	Accuracy	0.823	0.823	0.707
	p-value	< 2.2e-16	< 2.0e-16	< 2.0e-16

**Table 3:** Evaluation statistics for three methods (xgboost, random forest and neural network) for prediction of the postmortem interval, event location, and manner of death. The results for the three methods are put within the same table in the order of xgboost/ random forest/ neural network.

Predictor Variable	Performance Metric	< 24 h	25–48 h	49–72 h	> 73 h
**Postmortem Interval**	Sensitivity^1^	0.78/0.79/0.75	0.77/0.82/0.76	0.52/0.30/0.30	0.63/0.31/0.52
	Specificity^2^	0.78/0.79/0.79	0.80/0.76/0.77	0.99/0.99/0.97	0.99/1.00/0.98
	Pos Pred Value^3^	0.74/0.76/0.74	0.73/0.71/0.70	0.85/0.77/0.45	0.86/0.94/0.64
	Neg Pred Value^4^	0.81/0.82/0.80	0.83/0.85/0.82	0.96/0.94/0.94	0.98/0.96/0.97
	Prevalence^5^	0.45/0.45/0.45	0.42/0.42/0.42	0.08/0.08/0.08	0.06/0.06/0.06
	Detection Rate^6^	0.35/0.35/0.34	0.32/0.34/0.32	0.04/0.02/0.02	0.04/0.02/0.03
	Detection Prevalence^7^	0.47/0.47/0.45	0.44/0.48/0.45	0.05/0.03/0.05	0.04/0.02/0.04
	Balanced Accuracy^8^	0.78/0.79/0.77	0.78/0.79/0.77	0.75/0.65/0.64	0.81/0.66/0.75
**Event Location**		**Hospital**	**Indoors**	**Outdoors**	**Vehicular**
	Sensitivity	0.67/0.58/0.62	0.98/0.99/0.92	0.71/0.63/0.68	0.30/0.33/0.45
	Specificity	0.99/1.00/0.96	0.73/0.61/0.76	0.97/0.98/0.96	1.00/1.00/0.98
	Pos Pred Value	0.87/0.97/0.68	0.89/0.85/0.90	0.78/0.83/0.69	0.80/1.00/0.51
	Neg Pred Value	0.95/0.94/0.94	0.95/0.98/0.81	0.96/0.95/0.95	0.97/0.97/0.97
	Prevalence	0.13/0.13/0.13	0.70/0.70/0.70	0.13/0.13/0.13	0.05/0.05/0.05
	Detection Rate	0.09/0.08/0.08	0.68/0.69/0.64	0.09/0.08/0.09	0.01/0.02/0.02
	Detection Prevalence	0.10/0.08/0.12	0.77/0.81/0.71	0.11/0.10/0.12	0.02/0.02/0.04
	Balanced Accuracy	0.83/0.79/0.79	0.86/0.80/0.84	0.84/0.81/0.82	0.65/0.66/0.71
**Manner of Death**		**Accident**	**Homicide**	**Natural**	**Suicide**
	Sensitivity	0.84/0.88/0.79	0.88/0.87/0.74	0.86/0.90/0.69	0.60/0.38/0.43
	Specificity	0.87/0.87/0.80	0.98/0.99/0.94	0.90/0.88/0.88	0.98/0.99/0.96
	Pos Pred Value	0.81/0.82/0.72	0.93/0.96/0.75	0.78/0.75/0.70	0.84/0.89/0.57
	Neg Pred Value	0.89/0.92/0.85	0.97/0.97/0.94	0.94/0.96/0.87	0.95/0.92/0.92
	Prevalence	0.40/0.40/0.40	0.19/0.19/0.19	0.29/0.29/0.29	0.12/0.12/0.12
	Detection Rate	0.33/0.35/0.31	0.17/0.17/0.14	0.25/0.26/0.20	0.07/0.05/0.05
	Detection Prevalence	0.41/0.43/0.44	0.18/0.17/0.19	0.32/0.35/0.28	0.09/0.05/0.09
	Balanced Accuracy	0.85/0.87/0.79	0.93/0.93/0.84	0.88/0.89/0.79	0.79/0.69/0.69

^1^ Sensitivity: the proportion of positives that are correctly identified.

^2^ Specificity: the proportion of negatives that are correctly identified.

^3^ Pos Pred Value: proportions of positive results that are true positive.

^4^ Neg Pred Value: proportions of negative results that are true negative.

^5^ Prevalence: the proportion of a population who have a specific characteristic in a given time period.

^6^ Detection rate: the proportion of individuals with a particular condition who test positive for that condition when measured by some method.

^7^ Detection Prevalence: the proportion of the predicted events.

^8^ Balanced Accuracy: the average of the proportion of correct classifications within a class.

**Fig 1:**
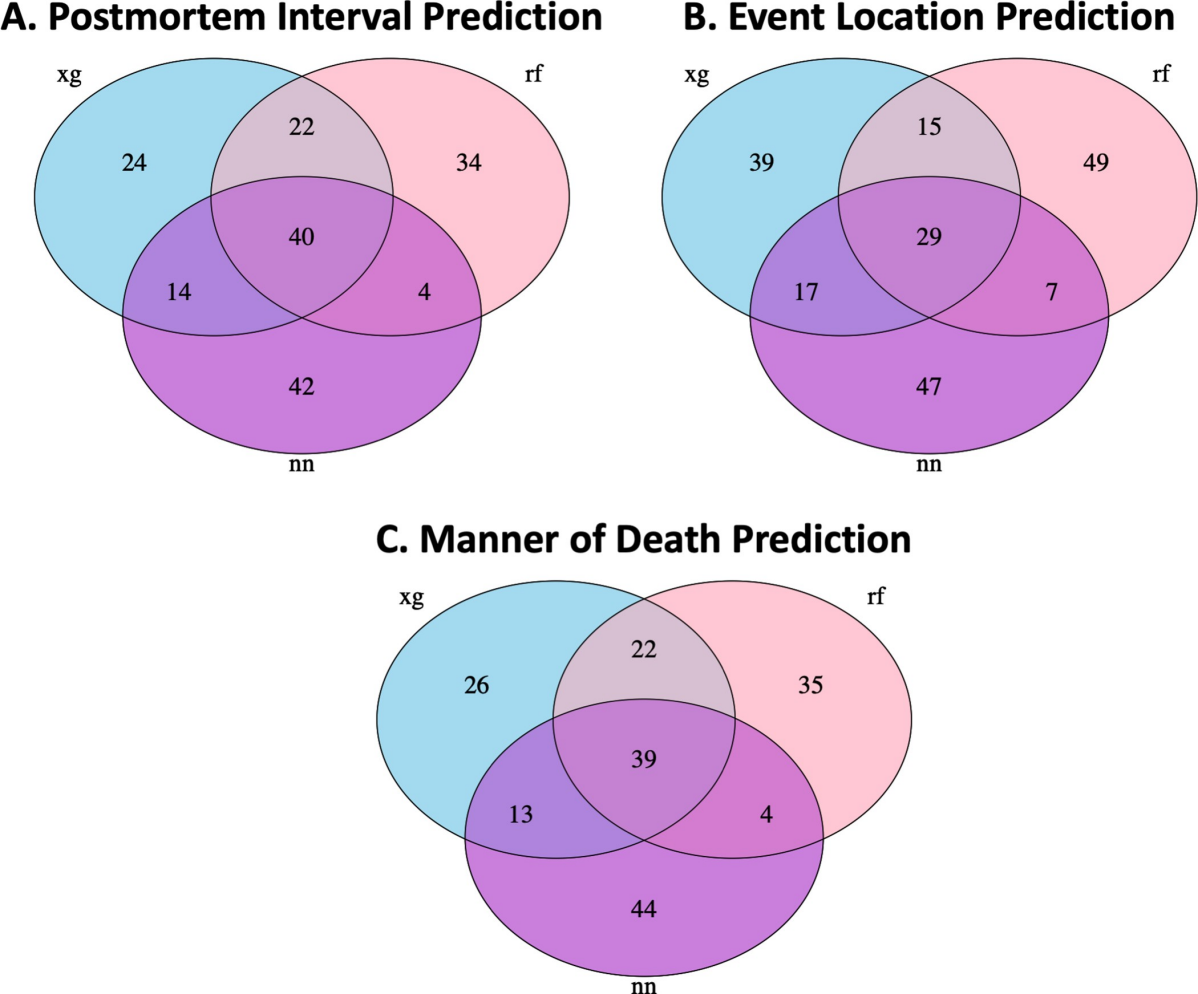
Common features among machine learning methods to predict case attributes. Venn diagram for shared features among top 100 features for predicting postmortem interval, event location, manner of death across three models xgboost (xg), random forest (rf), and neural network (nn). Identities of the features found by all methods can be found in S2–S5 Tables.

The top 100 most informative features were compared across methods to identify potentially important shared indicators (Fig 1, S2–S4 Tables). Overall, 40, 29, and 39 features were shared among all three methods (S5 Table) for postmortem interval estimate (Fig 1A), event location (Fig 1B), and manner of death (Fig 1C), respectively. Xgboost and random forest shared the greatest number of additional common features (22 for postmortem interval and 22 for manner of death), while neural network and random forest shared the least number of additional common features (4 for postmortem interval, 7 for event location, and 4 for manner of death). Analyzing the top ten important features for each method (there were eleven non-microbial metadata provided to the model), we found that all models only listed 1–3 microbes in the top ten features, with the rest of the top features being metadata (S2–S4 Tables).

### Predicting model performance based on anatomic area

**Fig 2:**
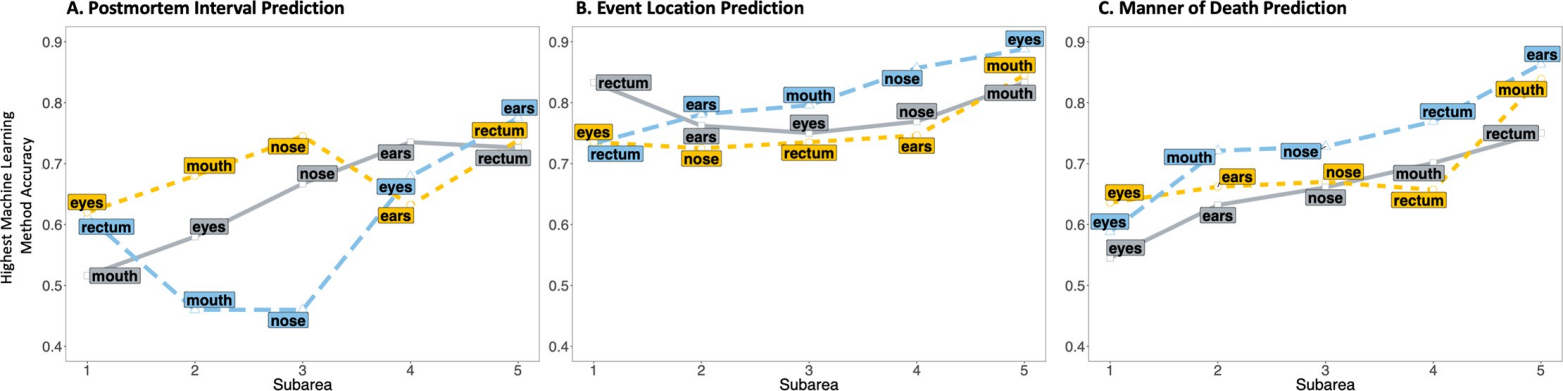
Method prediction accuracy based on number of anatomical areas. Prediction with all combinations of swabs for postmortem interval, event location and manner of death. Results for the most accurate model (highest accuracy) for a given number of samples from different subareas (anatomic areas). xgboost = blue dashed line; random forest = orange short dashed line; and neural network = gray solid line.

In the results described above, we ran full models that comprised five anatomic areas: ears, eyes, nose, mouth, and rectum. However, it is important to identify which anatomic area(s) provided the microbial community with the best predictive accuracy (Fig 2, Table 4). Using a greedy algorithm, we found that when predicting the postmortem interval (Fig 2A), the highest accuracy (77.5%) was achieved for xgboost when all five anatomic areas were used in the model; random forest had the highest accuracy (62–74.5%) with one to three anatomic areas (eye, mouth, nose); and neural network had the highest accuracy (73.5%) when a four anatomic area (eyes, mouth, ears, nose) model was implemented. For event location predictions (Fig 2B), the microbiota from all five anatomic areas were more accurate using xgboost (88.8% accuracy). For random forest, models with non-oral swabs provided generally the same (72.5–74.6%), but adding the oral swab increased accuracy in the full model to 84.5%. The method accuracy for predicting manner of death (Fig 2C) followed the trend that addition of a swab generally always improved predictions. Eyes were the most informative single swabs for all models predicting manner of death. Xgboost and neural networks increased accuracy with each swab (58.8–86.3 and 54.5–75.0%, respectively), whereas random forest again provided generally the same accuracy with 1–4 swabs (63.6–67%), with a stark increase to 83.9% when the oral swabs were included in the full model. In two cases (xgboost/PMI and neural network/Event location), the rectum provided high accuracy (comparable to a full model) as the sole source of microbiome information.

**Table 4:** Machine learning method accuracy from each model with a combination of subareas for the attribution predictor. The order of subareas (1 through 5) reflects the sequential addition and the respective accuracy when included in the model through the use of a greedy algorithm. “Subarea” is the microbiota from a specific anatomic area. The model with the highest accuracy within each method for each predictor variable is indicated with an asterisk (*).

Predictor Variable	Machine Learning Method	Subarea 1 (accuracy)	Subarea 2 (accuracy)	Subarea 3 (accuracy)	Subarea 4 (accuracy)	Subarea 5 (accuracy)
**Postmortem Interval Estimate**	xgboost	rectum (0.61)	mouth (0.46)	nose (0.46)	eyes (0.68)	ears* (0.78)
	random forest	eyes (0.62)	mouth (0.68)	nose* (0.75)	ears (0.63)	rectum (0.74)
	neural network	mouth (0.52)	eyes (0.58)	nose (0.67)	ears* (0.74)	rectum (0.73)
**Event Location**	xgboost	rectum (0.73)	ears (0.78)	mouth (0.80)	nose (0.86)	eyes* (0.89)
	random forest	eyes (0.74)	nose (0.73)	rectum (0.74)	ears (0.75)	mouth* (0.85)
	neural network	rectum* (0.83)	ears (0.76)	eyes (0.75)	nose (0.77)	mouth* (0.83)
**Manner of Death**	xgboost	eyes (0.59)	mouth (0.72)	nose (0.73)	rectum (0.77)	ears* (0.86)
	random forest	eyes (0.64)	ears (0.66)	nose (0.67)	rectum (0.66)	mouth* (0.84)
	neural network	eyes (0.55)	ears (0.63)	nose (0.66)	mouth (0.70)	rectum* (0.75)

### Important features

Based on metadata features (S1 Fig), the typical case for postmortem interval classes greater than 73 h postmortem tended to be older (> 55 years), male, and died of natural causes. Additionally, an increased number of cases occurred during the autumn and winter for those with an estimated postmortem interval greater than two days. The number of underweight (body mass index) cases peaked in cases with estimated postmortem intervals of 49–72 h.

Among the important microbial taxa identified for postmortem interval prediction (S2 Fig; S6 Table), we found *Veillonella dispar* sp. and *Proteus* sp. counts were higher in cases with a postmortem interval greater than 73 h, while Moraxellaceae had a higher count in cases with estimated postmortem intervals of 49–72 h, *Streptococcus* sp. count was higher within a 48 h postmortem interval. For event location prediction (S3 Fig; S6 Table), we found that Xanthomonadaceae was more prevalent in cases associated with hospital deaths. Suicide cases tended to have higher *Actinomyces* sp. counts than homicides, natural or accidental deaths (S4 Fig; S6 Table).

## Discussion

There is no best algorithm for making predictions with postmortem microbial data from this set of 188 death investigations with multiple samples per case. Inter-model accuracy depends on number and location of the microbial community on the body, in addition to, the type of predictor variables and question being asked. Different models use different features to make their predictions, with 29–40 of their top 100 features overlapping among all methods. It is not surprising that metadata categories are important predictors. For example, older, males that died of natural causes (e.g., cardiovascular disease) represent a common case with postmortem intervals after two days. However, from this dataset there are important bacterial predictors and the bacterial taxa in the shared list of most important features are promising for future investigation as predictors of forensic interest. One interesting observation in this analysis is how the importance of metadata differs across prediction variables (postmortem interval, event location, manner of death) and by machine learning method. Microbiota information appeared most useful in estimating postmortem interval. All models listed microbial features in their top ten features, though these ranked between the 6^th^ and 10^th^ most important and the first five features being metadata. The relative importance of these pieces of information, which will be particular to the demographics of any population, may be important to future dissection of the need for local versus global databases for such an endeavor.

In this analysis, xgboost generated the highest accuracy when incorporating information from all anatomical areas. The algorithm had high positive prediction values that generally increased with the number of anatomic area (microbial communities) included in the model. However, random forest sometimes performed better with lower numbers of anatomical areas in the model with higher sensitivity for less frequent classes (e.g., hospital cases, suicides, or estimated postmortem intervals of two to three days after death), hence it may be a method less sensitive to changes in additional anatomic area inclusion after the eyes, mouth, and nose. Differences between the performance outcomes of these two methods may be due to overfitting, which can be addressed in the future with validation studies using these or similar data as the reference database.

One observation noted here, which is supported by previous studies [6,9], is that most informative bacterial features are not represented in all samples and show distinct but weak trends on a taxon-to-taxon basis. For example, *Proteus* sp. exhibited higher occurrences in cases with estimated decomposition of more than two days. Members within the *Proteus* genus are characterized by a swarming behavior [27] and have been identified in longitudinal studies performed on human bodies decomposing at anthropological research facilities [28]. The microbial community signatures (measured here as individual taxon abundances in a sample) shift during the transition of less than to more than two days after death [6], with an increased number of unique taxa associated with communities within first two days after death. It has also been demonstrated that as decomposition progresses, postmortem microbial community richness and diversity significantly decreases after a postmortem interval of 48 hours or greater [6]. These previous observations in combination with the results from this machine learning analysis suggest that community information may be more useful than individual taxa. However, it remains possible that a combination of several weak predictors may still be equally or more informative of specific conditions, such as homicides or indoor deaths. Taxon targeted analyses may also be limited by the high incidence of absent taxa in any particular case, indicating the need to identify a variety of medical and forensic indicator biomarkers in order to ensure that at least one of them is found during investigation. Increased samples size and expansion of geographic location may help to inform if this observation is true across larger populations.

One critical finding in this analysis is the information provided by adding communities from multiple anatomical areas to the machine learning methods. The details of a particular case may not always allow for collection of swabs from all anatomical areas, highlighting the need for flexibility. This analysis provides some information about the expected quality of models when limited information (or budget for analysis) is available. For two prediction types, PMI and manner of death, adding sample locations usually increased accuracy models. For prediction of death location, improvements to accuracy were not as pronounced, though it is worth noting that this type of prediction was the most accurate overall. Curiously, sample location did not appear in the top 100 features of all but the neural network predicting the location of death, where it ranked 62nd (S2–S4 Tables). This lack of importance attributed to the variable suggests many microbial taxa provide similar information across sample sites. Yet, increasing the numbers of samples to make predictions generally increased (or did not harm) prediction accuracy and the most accurate predictions occurred with all swab locations included in the models. These results may indicate that the additional data from multiple swab sites on remains is more informative than the locations of the swabs. When making predictions with different anatomic area communities, the body sites that were more informative depended on the question being asked in this study: What is the time of death? Where was the location of death? or What was the manner of death? Indeed, in most cases analyzing more than three or four anatomical communities returned limited additional information. These observations make sense, as rectum and ear communities clustered separately and very distinctly in Pechal et al. [6], with oral and other communities clustering more widely and with greater overlap. Thus it makes sense that a few communities would be expected to provide the most generalizable information about a death. Providing one additional anatomical microbiota does seem to be informative, but two or three additional communities may not justify the cost of their inclusion. When evaluating this issue, the concerns with overfitting noted above must be considered in future research. Regardless of the microbiota from each anatomic area or machine learning method, this analysis provides some baseline information regarding the probable probative value of a particular type of sample to an investigation and in developing reference databases.

In the future, it may be possible to develop machine learning guided molecular autopsies taking advantage of either a local database curated by medical examiner’s offices, from regional databases or from one developed at a national level. Decisions regarding the cost-benefit analysis of such an endeavor will depend on the size of databases required to implement the predictions, the ability to collect microbiota from different anatomic areas, which will likely vary by budget across jurisdictions and by availability in specific cases, the desired prediction accuracy (or other metrics), and what investigators want to predict or estimate (e.g., postmortem interval vs. manner of death vs. undiagnosed medical conditions). A critical feature of such a system would include the machine learning method (or methods) implemented to make predictions and their performances in specific cases, which has been preliminarily addressed here. Additionally, the model performance comparisons shown here are also directly relevant to the development of future medical diagnostic platforms that evaluate human health conditions using postmortem microbiomes. While in its infancy, the potential for this new form of medical-laboratory method and its immediate diagnostic utility, as well as potential for public health surveillance, is gaining support with additional studies focused on the postmortem microbiome.

## Supporting information

S1 Data Data file for analyses. The data used in this analysis.
(ZIP)

S2 Data Code. A .zip file containing code to implement analyses reported here.
(ZIP)

S1 Fig Important metadata features to predict the estimated postmortem interval. The proportion of metadata was calculated for each class of the estimated postmortem interval (< 24 h, 25–48 h, 49–72 h, > 73 h). These metadata for each case included the (A) age in years, (B) sex, (C) manner of death, (D) season the death was reported, and (E) a categorized body mass index.
(TIFF)

S2 Fig Important microbiota features to predict the estimated postmortem interval. The left panel are boxplots of the raw count data while the right panel displays the ratio of non-zero to zero microbial taxon for potentially important microbial biomarkers (A: *Haemophilus parainfluenzae*, B: Moraxellaceae, C: *Veillonella dispar*, D: *Streptococcus* sp., E: *Proteus* sp.) in the estimated postmortem interval classes (< 24 h, 25–48 h, 49–72 h, > 73 h).
(TIFF)

S3 Fig Important microbiota features to predict the death event location. The left panel are boxplots of the raw count data while the right panel displays the ratio of non-zero to zero microbial taxon for potentially important microbial biomarkers (A: *Streptococcus* sp.; B: Xanthomonadaceae) in the event location classes (hospital, indoors, outdoors, vehicular).
(TIFF)

S4 Fig Important microbiota features to predict the manner of death. The left panel are boxplots of the raw count data while the right panel displays the ratio of non-zero to zero microbial taxon for potentially important microbial biomarkers (A: *Actinomyces* sp., B: *Haemophilus parainfluenzae*) in the manner of death classes (accident, homicide, natural, sucicide).
(TIFF)

S1 Table Tuning parameters. Tuning parameters tested to determine optimal choices for subsequent analyses.
(DOCX)

S2 Table Top postmortem interval features. Top 100 features to predict the estimated postmortem interval.
(DOCX)

S3 Table Top event location features. Top 100 features to predict the event location.
(DOCX)

S4 Table Top manner of death features. Top 100 features to predict the manner of death.
(DOCX)

S5 Table Shared top features. Shared features across all three machine learning methods to predict case attributes.
(DOCX)

S6 Table Statistical comparison of important features. Pairwise Wilcox rank sum test of important features.
(DOCX)
