# Supplementary figures and images for: Machine learning performance in a microbial molecular autopsy context: A cross-sectional postmortem human population study
Source: PLoS One. 2019 Apr 15;14(4):e0213829. doi: 10.1371/journal.pone.0213829 (PMC6464165; doi:10.1371/journal.pone.0213829)

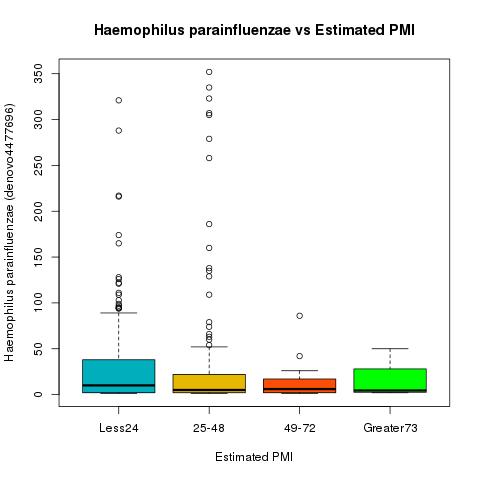

Supplement: S2 Data — A .zip file containing code to implement analyses reported here. (ZIP) [file pone.0213829.s002.zip › Zhang_et_al/output/fig_S2A1.png]

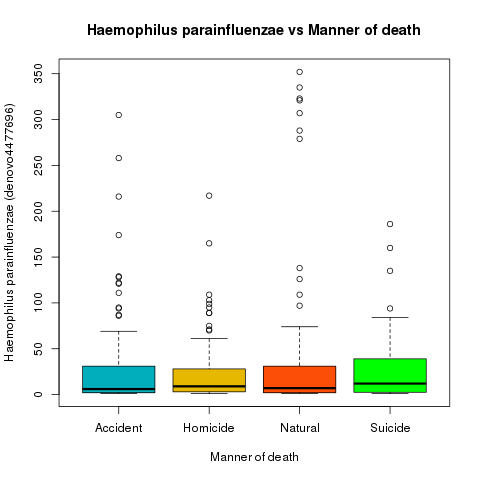

Supplement: S2 Data — A .zip file containing code to implement analyses reported here. (ZIP) [file pone.0213829.s002.zip › Zhang_et_al/output/fig_S4B1.png]

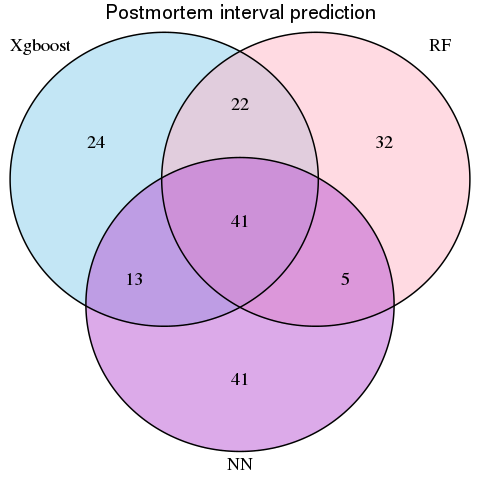

Supplement: S2 Data — A .zip file containing code to implement analyses reported here. (ZIP) [file pone.0213829.s002.zip › Zhang_et_al/output/fig_1A.png]

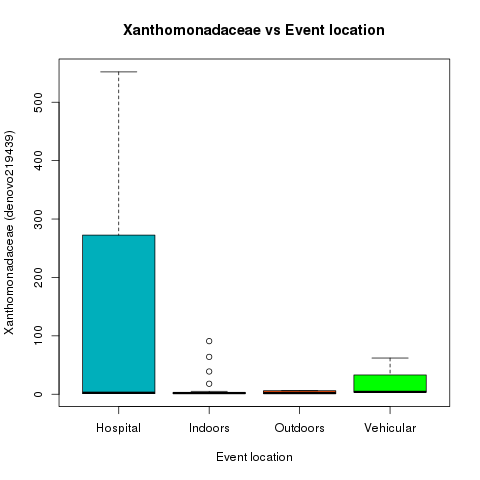

Supplement: S2 Data — A .zip file containing code to implement analyses reported here. (ZIP) [file pone.0213829.s002.zip › Zhang_et_al/output/fig_S3B1.png]

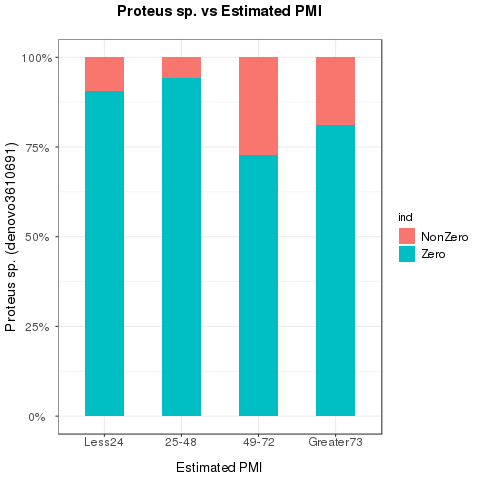

Supplement: S2 Data — A .zip file containing code to implement analyses reported here. (ZIP) [file pone.0213829.s002.zip › Zhang_et_al/output/fig_S2C2.png]

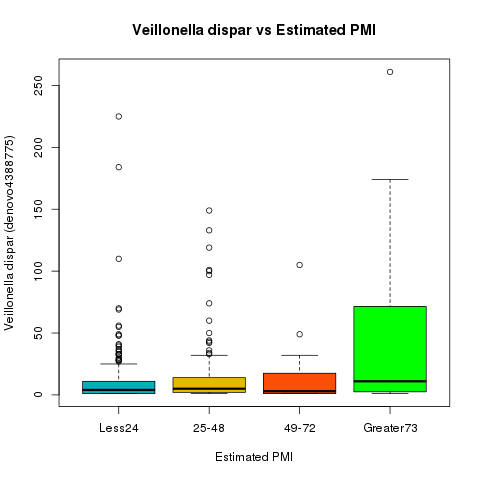

Supplement: S2 Data — A .zip file containing code to implement analyses reported here. (ZIP) [file pone.0213829.s002.zip › Zhang_et_al/output/fig_S2B1.png]

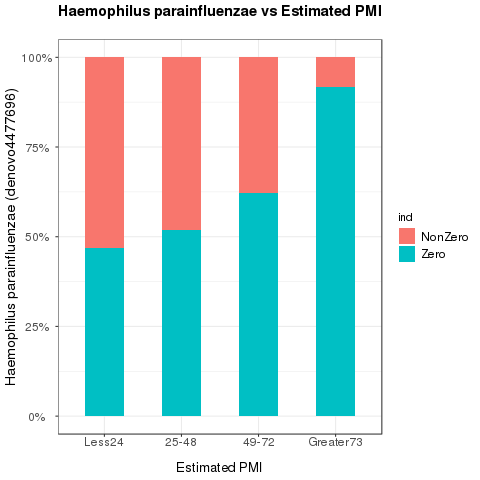

Supplement: S2 Data — A .zip file containing code to implement analyses reported here. (ZIP) [file pone.0213829.s002.zip › Zhang_et_al/output/fig_S2A2.png]

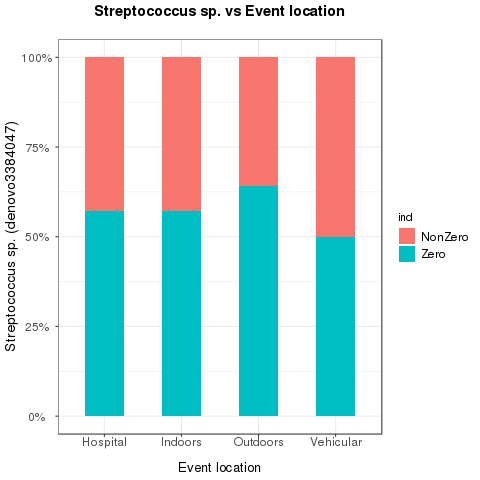

Supplement: S2 Data — A .zip file containing code to implement analyses reported here. (ZIP) [file pone.0213829.s002.zip › Zhang_et_al/output/fig_S3A2.png]

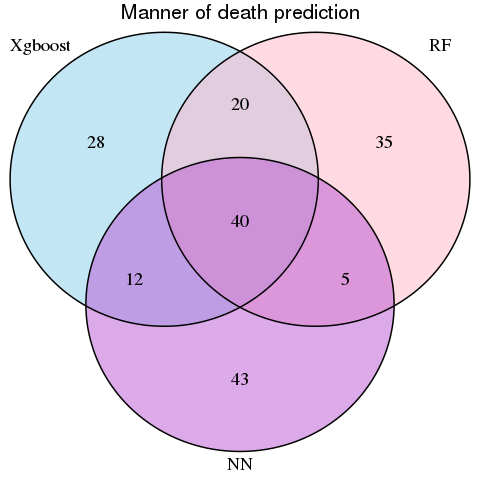

Supplement: S2 Data — A .zip file containing code to implement analyses reported here. (ZIP) [file pone.0213829.s002.zip › Zhang_et_al/output/fig_1C.png]

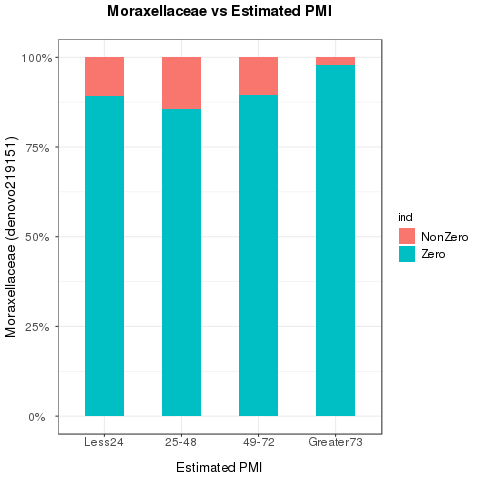

Supplement: S2 Data — A .zip file containing code to implement analyses reported here. (ZIP) [file pone.0213829.s002.zip › Zhang_et_al/output/fig_S2D2.png]

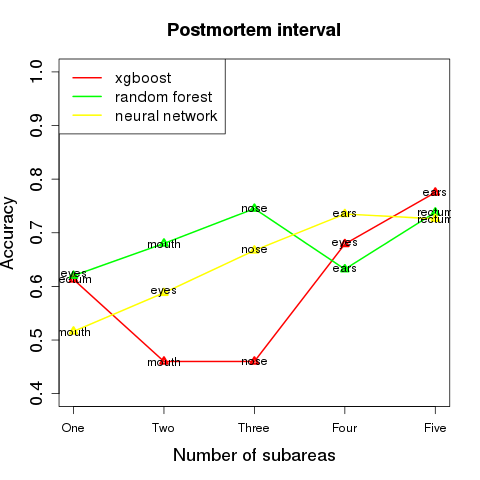

Supplement: S2 Data — A .zip file containing code to implement analyses reported here. (ZIP) [file pone.0213829.s002.zip › Zhang_et_al/output/fig_2A.png]

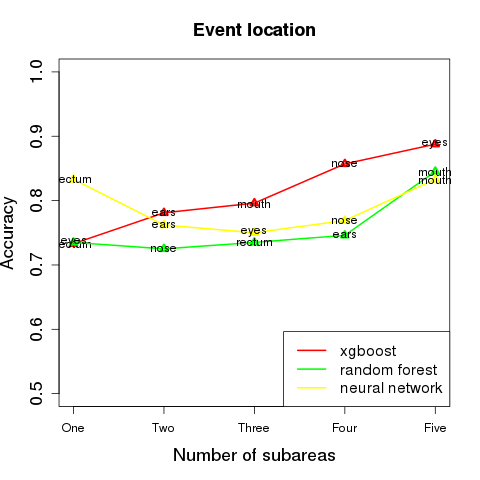

Supplement: S2 Data — A .zip file containing code to implement analyses reported here. (ZIP) [file pone.0213829.s002.zip › Zhang_et_al/output/fig_2B.png]

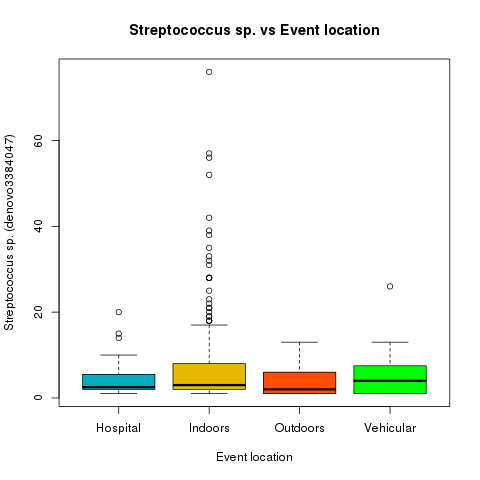

Supplement: S2 Data — A .zip file containing code to implement analyses reported here. (ZIP) [file pone.0213829.s002.zip › Zhang_et_al/output/fig_S3A1.png]

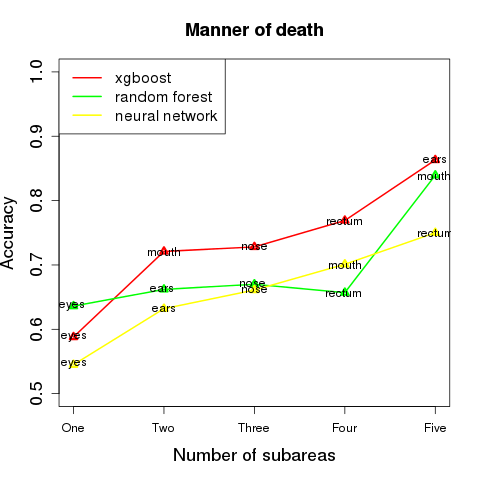

Supplement: S2 Data — A .zip file containing code to implement analyses reported here. (ZIP) [file pone.0213829.s002.zip › Zhang_et_al/output/fig_2C.png]

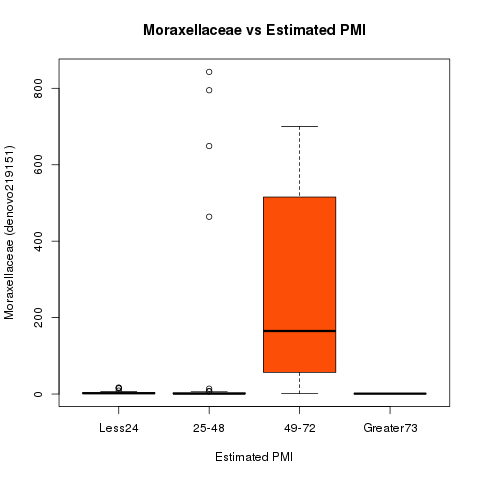

Supplement: S2 Data — A .zip file containing code to implement analyses reported here. (ZIP) [file pone.0213829.s002.zip › Zhang_et_al/output/fig_S2D1.png]

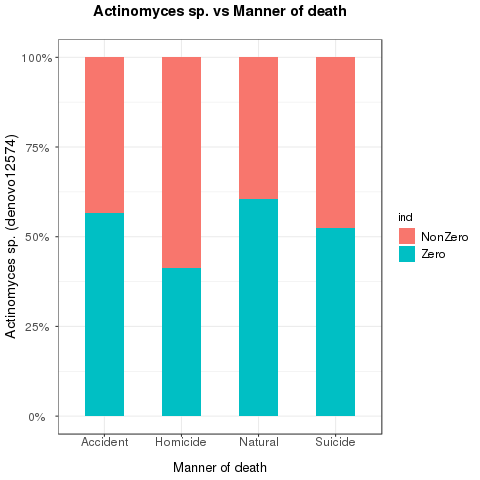

Supplement: S2 Data — A .zip file containing code to implement analyses reported here. (ZIP) [file pone.0213829.s002.zip › Zhang_et_al/output/fig_S4A2.png]

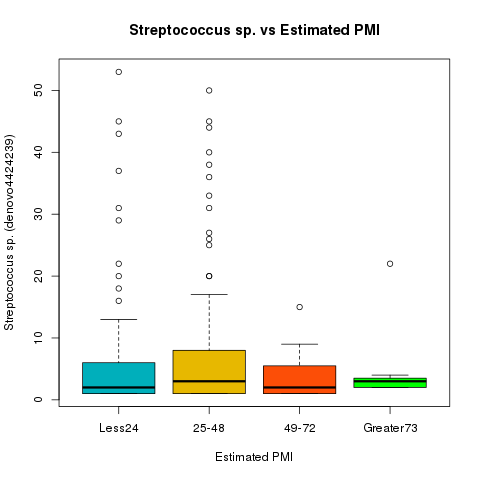

Supplement: S2 Data — A .zip file containing code to implement analyses reported here. (ZIP) [file pone.0213829.s002.zip › Zhang_et_al/output/fig_S2E1.png]

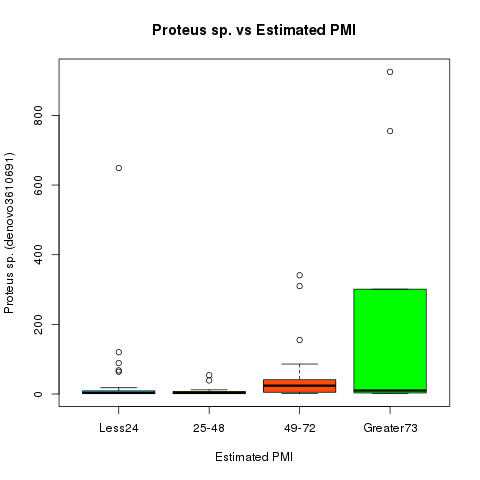

Supplement: S2 Data — A .zip file containing code to implement analyses reported here. (ZIP) [file pone.0213829.s002.zip › Zhang_et_al/output/fig_S2C1.png]

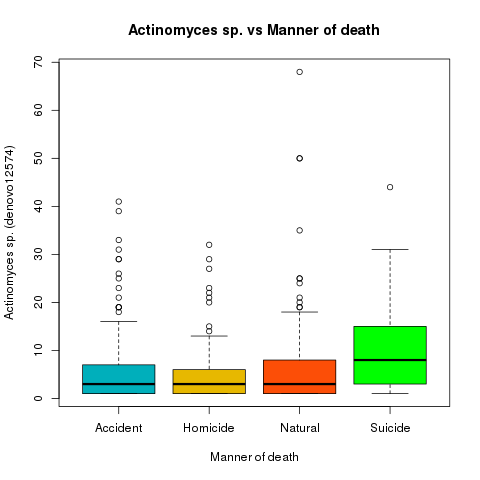

Supplement: S2 Data — A .zip file containing code to implement analyses reported here. (ZIP) [file pone.0213829.s002.zip › Zhang_et_al/output/fig_S4A1.png]

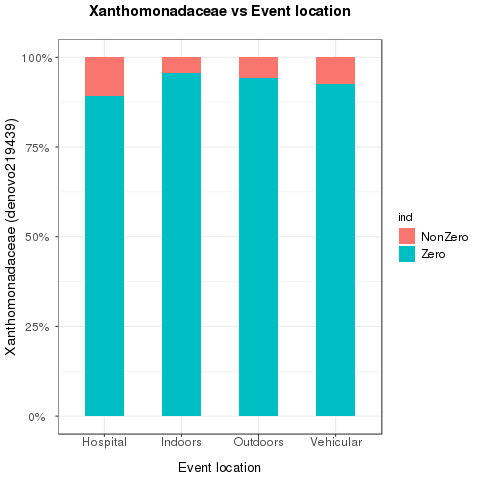

Supplement: S2 Data — A .zip file containing code to implement analyses reported here. (ZIP) [file pone.0213829.s002.zip › Zhang_et_al/output/fig_S3B2.png]

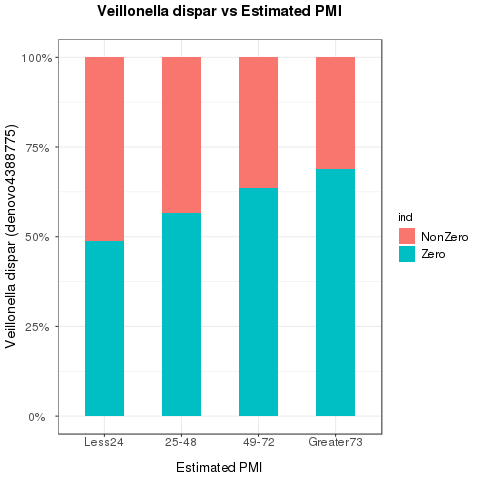

Supplement: S2 Data — A .zip file containing code to implement analyses reported here. (ZIP) [file pone.0213829.s002.zip › Zhang_et_al/output/fig_S2B2.png]

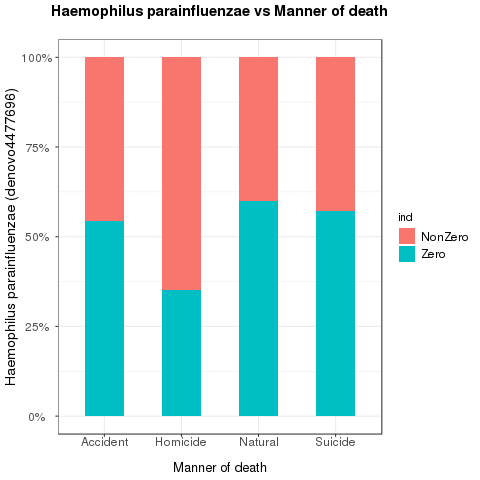

Supplement: S2 Data — A .zip file containing code to implement analyses reported here. (ZIP) [file pone.0213829.s002.zip › Zhang_et_al/output/fig_S4B2.png]

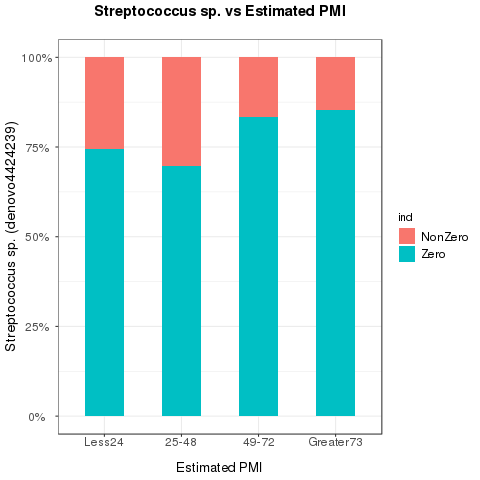

Supplement: S2 Data — A .zip file containing code to implement analyses reported here. (ZIP) [file pone.0213829.s002.zip › Zhang_et_al/output/fig_S2E2.png]

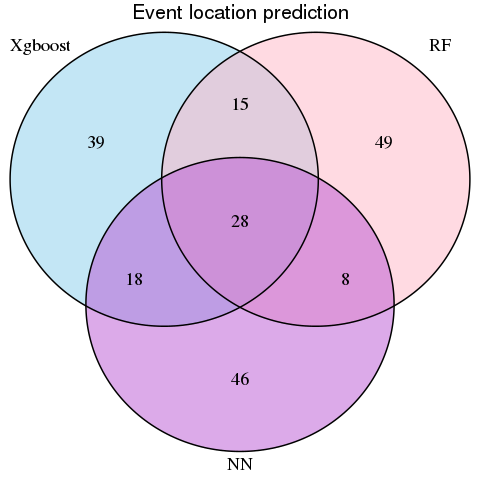

Supplement: S2 Data — A .zip file containing code to implement analyses reported here. (ZIP) [file pone.0213829.s002.zip › Zhang_et_al/output/fig_1B.png]

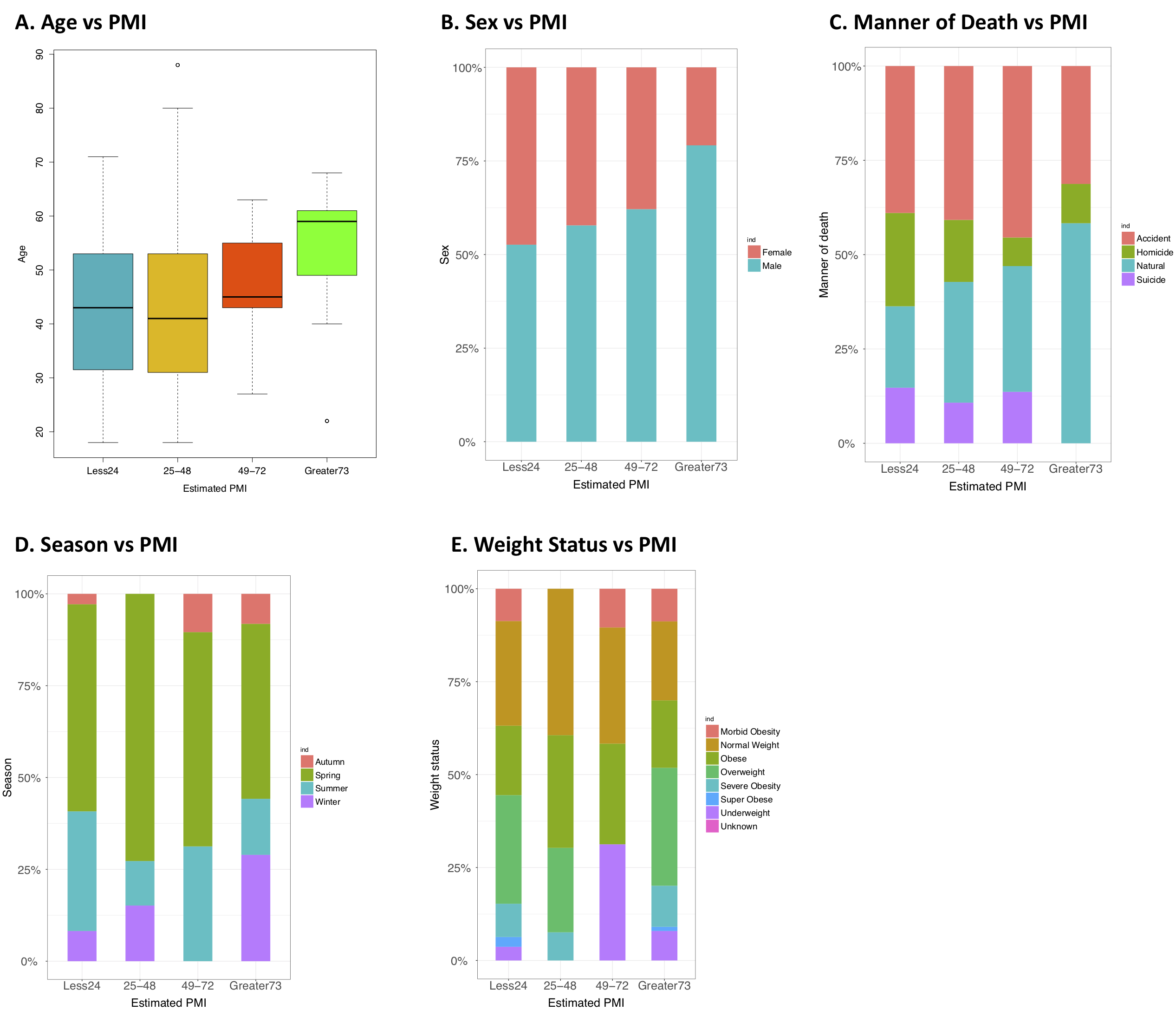

Supplement: S1 Fig — The proportion of metadata was calculated for each class of the estimated postmortem interval (< 24 h, 25–48 h, 49–72 h, > 73 h). These metadata for each case included the (A) age in years, (B) sex, (C) manner of death, (D) season the death was reported, and (E) a categorized body mass index. (TIFF) [file pone.0213829.s003.tiff]

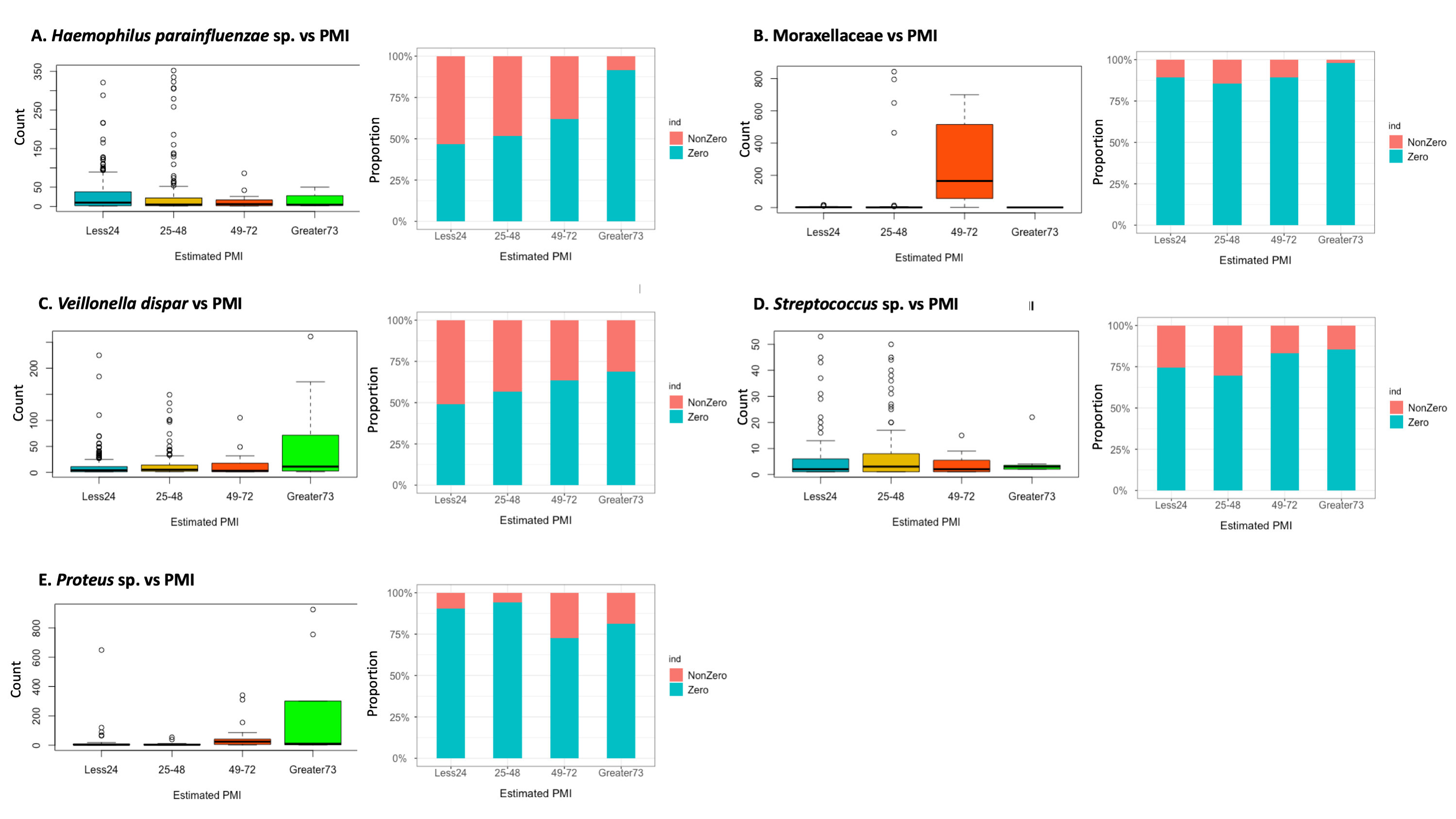

Supplement: S2 Fig — The left panel are boxplots of the raw count data while the right panel displays the ratio of non-zero to zero microbial taxon for potentially important microbial biomarkers (A: Haemophilus parainfluenzae, B: Moraxellaceae, C: Veillonella dispar, D: Streptococcus sp., E: Proteus sp.) in the estimated postmortem interval classes (< 24 h, 25–48 h, 49–72 h, > 73 h). (TIFF) [file pone.0213829.s004.tiff]

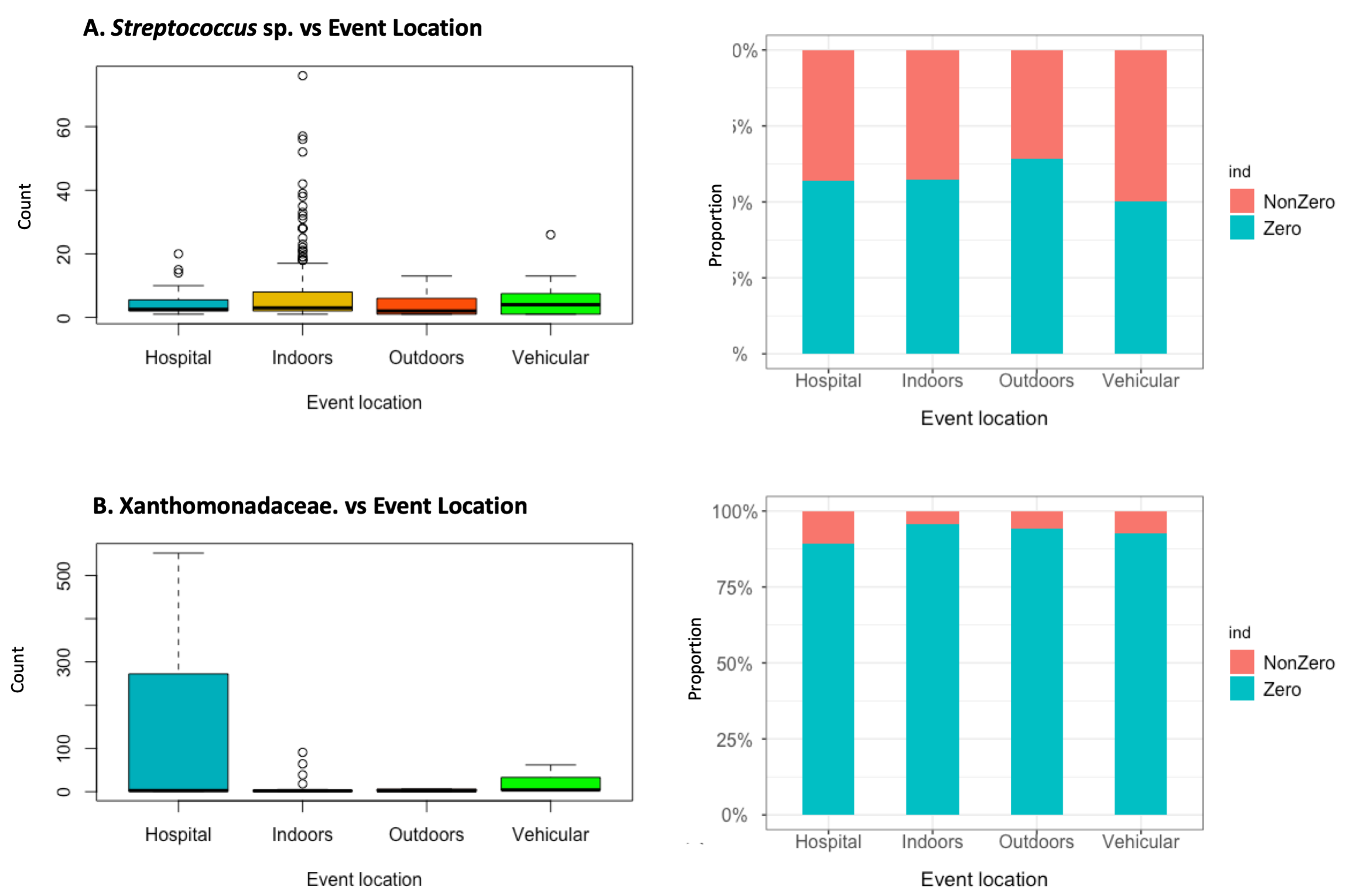

Supplement: S3 Fig — The left panel are boxplots of the raw count data while the right panel displays the ratio of non-zero to zero microbial taxon for potentially important microbial biomarkers (A: Streptococcus sp.; B: Xanthomonadaceae) in the event location classes (hospital, indoors, outdoors, vehicular). (TIFF) [file pone.0213829.s005.tiff]

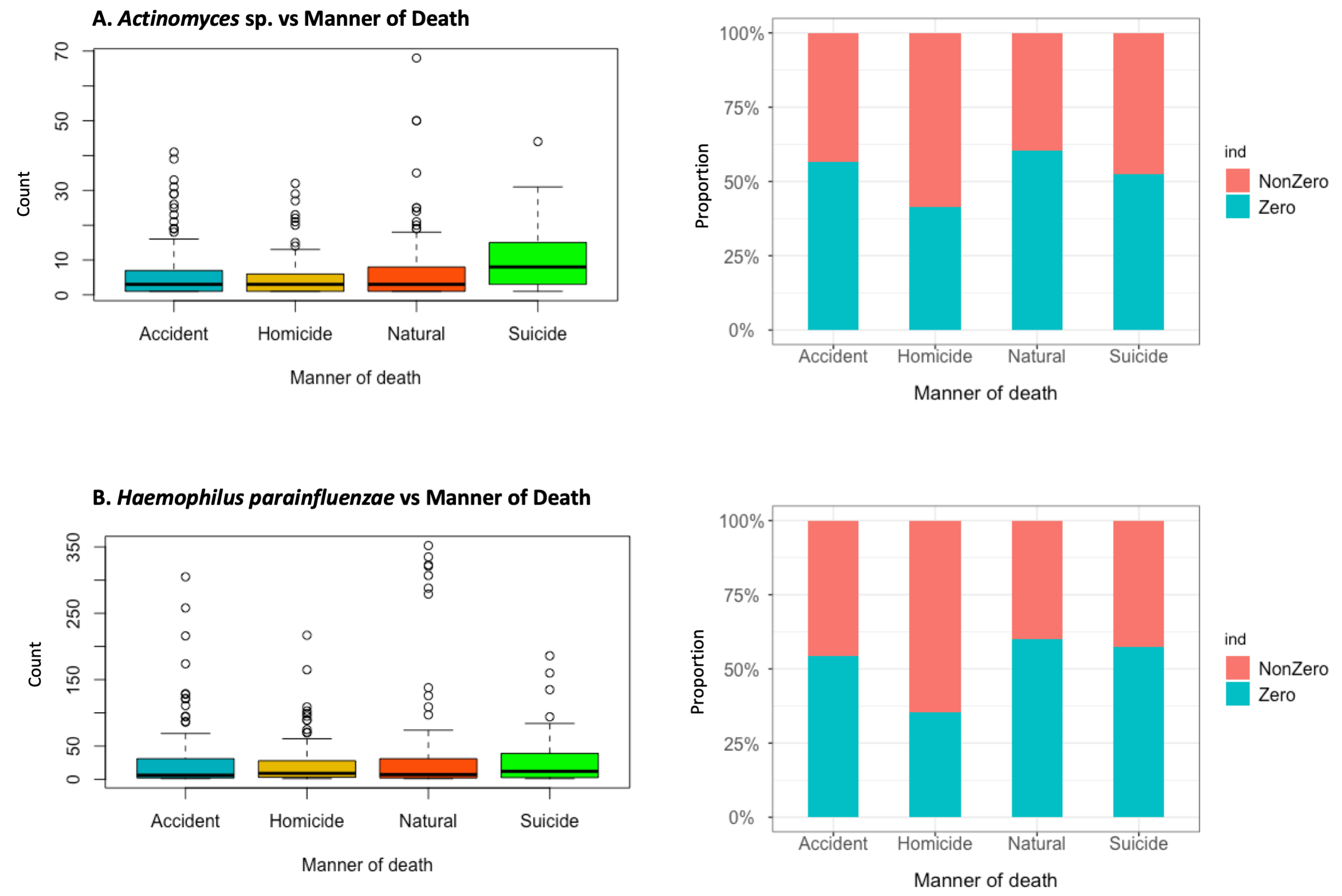

Supplement: S4 Fig — The left panel are boxplots of the raw count data while the right panel displays the ratio of non-zero to zero microbial taxon for potentially important microbial biomarkers (A: Actinomyces sp., B: Haemophilus parainfluenzae) in the manner of death classes (accident, homicide, natural, sucicide). (TIFF) [file pone.0213829.s006.tiff]
