# Supplementary material for: Machine learning performance in a microbial molecular autopsy context: A cross-sectional postmortem human population study
Source: PLoS One. 2019 Apr 15;14(4):e0213829. doi: 10.1371/journal.pone.0213829 (PMC6464165; doi:10.1371/journal.pone.0213829)
Supplement: S1 Table — Tuning parameters tested to determine optimal choices for subsequent analyses. (DOCX) [file pone.0213829.s007.docx]

**S1 Table**. Tuning parameters for (A) xgboost, (B) random forest, and (C) neural network for predicting estimated postmortem interval, event location and manner of death through 5-fold cross-validation.

| **Machine Learning Method** | **Predictor Variable** | **nround** | **eta** | **mtry** | **ntrees** | **size** | **decay** | **maxit** |
| --- | --- | --- | --- | --- | --- | --- | --- | --- |
| xgboost^1^ | Postmortem Interval | 900 | 0.2 | - | - | - | - | - |
|  | Event Location | 300 | 0.1 | - | - | - | - | - |
|  | Manner of Death | 1200 | 0.1 | - | - | - | - | - |
| random forest^2^ | Postmortem Interval | - | - | 1024 | 100 | - | - | - |
|  | Event Location | - | - | 512 | 100 | - | - | - |
|  | Manner of Death | - | - | 512 | 100 | - | - | - |
| neural network^3^ | Postmortem Interval | - | - | - | - | 6 | 0.2 | 250 |
|  | Event Location | - | - | - | - | 6 | 0.01 | 1000 |
|  | Manner of Death | - | - | - | - | 6 | 0.2 | 500 |

^1^ parameters set: nround = c(100, 200, 300, 400, 500, 600, 700, 800, 900, 1000, 1200, 1300, 1400, 1500), eta = c(0.1, 0.2)

^2^ parameters set: mtry = c(32, 64, 128, 256, 512, 1024), eta = c(100, 200, 300, 400, 500)

^3^ parameters set: size = c(3, 4, 5, 6), decay = c(0.001, 0.01, 0.1, 0.2, 0.5), maxit = c(250, 500, 1000)
