# Supplementary material for: Machine learning performance in a microbial molecular autopsy context: A cross-sectional postmortem human population study
Source: PLoS One. 2019 Apr 15;14(4):e0213829. doi: 10.1371/journal.pone.0213829 (PMC6464165; doi:10.1371/journal.pone.0213829)
Supplement: S6 Table — Pairwise Wilcox rank sum test of important features. (DOCX) [file pone.0213829.s012.docx]

**S6 Table**. Pairwise comparisons using Wilcoxon rank sum of important indicator taxa identified in S2-S4 Figures. Significant results indicated by an asterisk(s): * = false discovery rate < 0.05; ** = false discovery rate < 0.01; *** false discovery rate < 0.001.

| **Metadata** | **Taxon** |  |  |  |  |
| --- | --- | --- | --- | --- | --- |
| Postmortem Interval | *Haemophilus parainfluenzae* |  | **25-48 h** | **49-72 h** | **> 73 h** |
|  |  | **49-72** | 0.984 | - | - |
|  |  | **> 73 h** | 0.984 | 0.984 | - |
|  |  | **< 24 h** | 0.044* | 0.215 | 0.984 |
|  | *Veillonella dispar* |  | **25-48 h** | **49-72 h** | **> 73 h** |
|  |  | **49-72** | 0.370 | - | - |
|  |  | **> 73 h** | 0.180 | 0.180 | - |
|  |  | **< 24 h** | 0.120 | 0.760 | 0.120 |
|  | *Proteus* sp. |  | **25-48 h** | **49-72 h** | **> 73 h** |
|  |  | **49-72** | 0.014* | - | - |
|  |  | **> 73 h** | 0.230 | 0.637 | - |
|  |  | **< 24 h** | 0.637 | 0.014* | 0.230 |
|  | Moraxellaceae |  | **25-48 h** | **49-72 h** | **> 73 h** |
|  |  | **49-72** | 0.087 | - | - |
|  |  | **> 73 h** | 0.257 | 0.253 | - |
|  |  | **< 24 h** | 0.691 | 0.087 | 0.253 |
|  | *Streptococcus* sp. |  | **25-48 h** | **49-72 h** | **> 73 h** |
|  |  | **49-72** | 0.670 | - | - |
|  |  | **> 73 h** | 0.810 | 0.670 | - |
|  |  | **< 24 h** | 0.670 | 0.930 | 0.670 |
| **Event Location** |  |  |  |  |  |
|  | *Streptococcus* sp. |  | **Hospital** | **Indoors** | **Outdoors** |
|  |  | **Indoors** | 0.068 | - | - |
|  |  | **Outdoors** | 0.853 | 0.040* | - |
|  |  | **Vehicular** | 0.538 | 0.659 | 0.538 |
|  | Xanthomonadaceae |  | **Hospital** | **Indoors** | **Outdoors** |
|  |  | **Indoors** | 0.430 | - | - |
|  |  | **Outdoors** | 0.770 | 0.770 | - |
|  |  | **Vehicular** | 0.770 | 0.430 | 0.430 |
| **Manner of Death** |  |  |  |  |  |
|  | *Actinomyces* sp. |  | **Accident** | **Homicide** | **Natural** |
|  |  | **Homicide** | 0.611 | - | - |
|  |  | **Natural** | 0.913 | 0.611 | - |
|  |  | **Suicide** | 0.002** | 0.001** | 0.004** |
|  | *Haemophilus parainfluenzae* |  | **Accident** | **Homicide** | **Natural** |
|  |  | **Homicide** | 0.670 | - | - |
|  |  | **Natural** | 0.670 | 0.800 | - |
|  |  | **Suicide** | 0.670 | 0.670 | 0.670 |
